# Clinical value of serum Klotho protein in patients with acute traumatic brain injury complicated by acute kidney injury

**DOI:** 10.1038/s41598-025-05201-y

**Published:** 2025-07-01

**Authors:** Bin Ren, Jifang Liang, Junkun Zhang, Leifang Yang, Xiaocong Wei, Min Guo, Weidong Wu, Xinmin Ding

**Affiliations:** 1 Department of Neurosurgery, Shanxi Bethune Hospital, Shanxi Academy of Medical Sciences, Taiyuan, China; 2 Department of Intensive Care Unit, Shanxi Bethune Hospital, Shanxi Academy of Medical Sciences, Taiyuan, China; 3 Tongji Shanxi Hospital, Third Hospital of Shanxi Medical University, Taiyuan, 030032 China; 4 Shanxi Bethune Hospital, Shanxi Academy of Medical Sciences, Tongji Shanxi Hospital, Third Hospital of Shanxi Medical University, Taiyuan, 030032 China; 5 Department of Neurosurgery, Shanxi Bethune Hospital, Tongji Shanxi Hospital, Shanxi Academy of Medical Sciences, Third Hospital of Shanxi Medical University, Taiyuan, 030032 China

**Keywords:** Traumatic brain injury (TBI), Acute kidney injury (AKI), Prognostic marker, Klotho protein, Neuroscience, Brain injuries

## Abstract

Traumatic Brain Injury (TBI) is a major cause of mortality and morbidity, with acute kidney injury (AKI) as a common complication. This study aimed to investigate the potential of serum Klotho protein levels as a diagnostic and prognostic marker for AKI in TBI patients.The results showed that serum Klotho level 177.3 (165.43,195.07) (pg/ml) was elevated in patients with traumatic brain injury as compared to normal group 134.8(125.6,138.9) (pg/ml) . We conducted univariate analyses of all potential factors, resulting in four variables for inclusion in the multivariate analysis. Among them, the HR value of Klotho protein was 2.076. Comparing the predictive efficacy of serum creatinine and Klotho protein, the ROC value of Klotho protein was 0.832 (95% CI: 0.709–0.898), suggesting higher predictive ability than serum creatinine. Modestly elevated serum Klotho protein levels were associated with a higher risk of AKI and a poor long-term prognosis.These findings suggested that serum Klotho protein levels may serve as an early diagnostic indicator and predictor of outcomes in TBI patients with AKI, providing insights for potential therapeutic interventions. Further research is needed to validate these findings and explore the clinical utility of targeting the Klotho pathway in TBI-associated AKI.

## Introduction

Traumatic brain injury (TBI) is a major global health issue that has a considerable impact on morbidity and death rates, especially in people under the age of 40^1–3^ TBI is one of the most common causes of death in this age group, accounting for half a million cases each year. Acute kidney injury (AKI), which has a morbidity rate of about 20%, frequently occurs in individuals with craniocerebral injuries^4,5^. Significantly higher fatality rates are linked to AKI in these patients, especially in the critical care unit, where rates can range from 37 to 76%. As a result, AKI in patients with craniocerebral injuries causes significant social and economic consequences and demands clinician’s full attention^5,6^.

Klotho proteins, which come in forms that resemble membranes and secretory forms, have a variety of biological functions^1,7^. The membrane-type Klotho protein, which participates in calcium and phosphorus metabolism, functions as a co-receptor for fibroblast growth factor 23 (FGF23)^8^. With numerous physiologic consequences, the released Klotho protein acts as a humoral factor. Klotho has anti-inflammatory, antioxidant, and tubular protective effects by regulating multiple cell signaling pathways^9,10^. Klotho deficiency leads to apoptosis and increased oxidative stress in renal tubular cells, which is particularly evident in acute kidney injury. Studies have shown that Klotho protein levels are significantly decreased in patients with AKI, suggesting a possible important role of Klotho in the mechanism of AKI. In addition, the interaction of Klotho with fibroblast growth FGF23 is also thought to have an important impact on the progression of AKI, and Klotho supplementation has been found to attenuate the pathologic changes of AKI. Therefore, monitoring of Klotho levels may provide new biomarkers for early diagnosis and prognosis of AKI^11^.

However, only a few studies have looked at the clinical significance of changes in serum Klotho protein following AKI brought on by craniocerebral injury^1,12,13^. Therefore, it was necessary to investigate the connection between serum Klotho protein levels and the degree of brain damage in TBI patients as well as its prognostic significance for the development of AKI. These studies aimed to contribute to the early diagnosis and care of kidney impairment by identifying new clinical therapeutic targets. Recently, it has become clear that the FGF23/Klotho signaling pathway is an important target for the early diagnosis and treatment of kidney impairment^13^. FGF23, which is largely produced and secreted by osteocytes and osteoblasts^14^, is essential for the metabolism of calcium and phosphorus. Experimental research has shown that mice lacking FGF23 exhibit hyperphosphatemia, hypercalcemia, ectopic calcification, and osteoporosis, whereas mice overexpressing FGF23 exhibit hypophosphatemia, rickets, and osteomalacia^15^. The interaction of FGF23 receptors and Klotho proteins serve as the primary mechanism by which the FGF23/Klotho signaling pathway mediates biological signaling. Notably, patients with chronic kidney damage had considerably lower levels of Klotho mRNA and protein expression, pointing to the protein’s potential significance in kidney repair and injury development. The membrane-bound Klotho protein functions as a co-factor for FGF23, allowing FGF23/Klotho signalling to control renal tubular epithelial cell activity and control calcium and phosphorus metabolism^16^ In individuals with chronic renal injury, dysregulation of this signaling pathway causes problems with calcium and phosphorus balance.

In light of the aforementioned factors, the goal of this study was to examine the connection between traumatic brain injury severity and serum Klotho protein levels in TBI patients. We have also evaluated how well serum Klotho protein levels predict the development of AKI.

We hope to find potential therapeutic targets and improve the early diagnosis and care of kidney injury by understanding the clinical implications of serum Klotho protein alterations in this setting. In the end, this research might help in the management of craniocerebral injuries by improving patient outcomes.

## Methods

To better understand the blood levels of soluble Klotho protein in patients with craniocerebral trauma, a study was done at the Department of Critical Care Medicine at Shanxi Bethune Hospital.

There were no discernible differences between the patient and control groups in terms of their general characteristics. The inclusion criteria^17^ required that patients be admitted to the hospital within 24 h of the accident and have a confirmed diagnosis of a craniocerebral injury. Participants were required to provide complete clinical information, give informed consent, and be at least 18 years old. A history of cerebral infarction, cerebral hemorrhage, transient ischemic attack, chronic kidney disease, hypertension, diabetes mellitus, or malignant tumors was one of the exclusion criteria.

The KLOTHO ELISA kit (Cat. No.: EK1688) from Wuhan Boster Biological Engineering Co., Ltd. was used in the investigation to measure the concentrations of serum-soluble klotho protein^18^. The cubital venous puncture was used to obtain 3 mL of blood from both patients and healthy subjects. The supernatant was centrifuged at 3500 rpm for 10 min, and then it was frozen at -80 °C for further analysis.

Using the ELISA kit, trained inspectors consistently carried out the detection. Clinical information was gathered from the hospital’s information system’s storage of patient’s medical records with craniocerebral injuries. Name, hospitalization number, gender, age, etiology, treatment method, Glasgow Coma Scale (GCS) score, predicted mortality, and other pertinent clinical indicators, such as hemoglobin (Hb), white blood cell count (WBC), platelet count (PLT), and glucose (Glu) level, were all included in the database. The hospital’s laboratory division examined each clinical test signal. According to the 2012 KIDGO criteria, patients were divided into four groups according to whether they had acute kidney injury (AKI) or three different stages of AKI and intergroup comparisons of serum Klotho protein at different stages of renal function.

To evaluate the patient’s prognosis, follow-up was done over 90 days. Prognosis was assessed using the Glasgow Outcome Scale (GOS) score, and patients were divided into four groups to assess the differences in Klotho protein between the different groups. The mortality group consisted of patients who voluntarily departed the hospital due to death or a worsening of their condition, whereas the survival group consisted of patients whose health improved and who were either discharged or transferred to a general ward.

GraphPad Prism (version 8) and SPSS 22.0 were the two software programs used to conduct the statistical analysis. T-tests were used to assess the data, which came with the mean and standard deviation. The median and quartiles [M (P25, P75)] of non-normally distributed data were displayed using the Mann-Whitney U test or the Kruskal-Wallis test^18^. Those variables with *P* < 0.05 were candidates for multivariate logistic regression models. Candidate variables were entered into stepwise multivariate logistic regression using a backward selection model. The odds ratio (OR) and 95% confidence interval (CI) were obtained. ROC curves were used to compare the predictive value of Klotho protein levels and serum creatinine. The best cutoff point of Klotho protein were obtained using the Youden index. The statistical significance was defined as a P value of 0.05, based on two-tailed tests.The Ethics Committee of Shanxi Bethune Hospital granted ethical permission, and the patients’ and their families’ informed consent was obtained.

## Results

The study focused on the occurrence of acute kidney damage (AKI) and its correlation with serum Klotho protein levels in patients with craniocerebral injury. There were 122 patients in total—85 men and 37 females, ranging in age from 18 to 62—that were included in the study. A control group of 100 healthy volunteers was also assembled among those who received physical examinations at the same hospital. A control group consisted of 63 men and 37 women, ages ranging from 24 to 62.

**Fig. 1:**
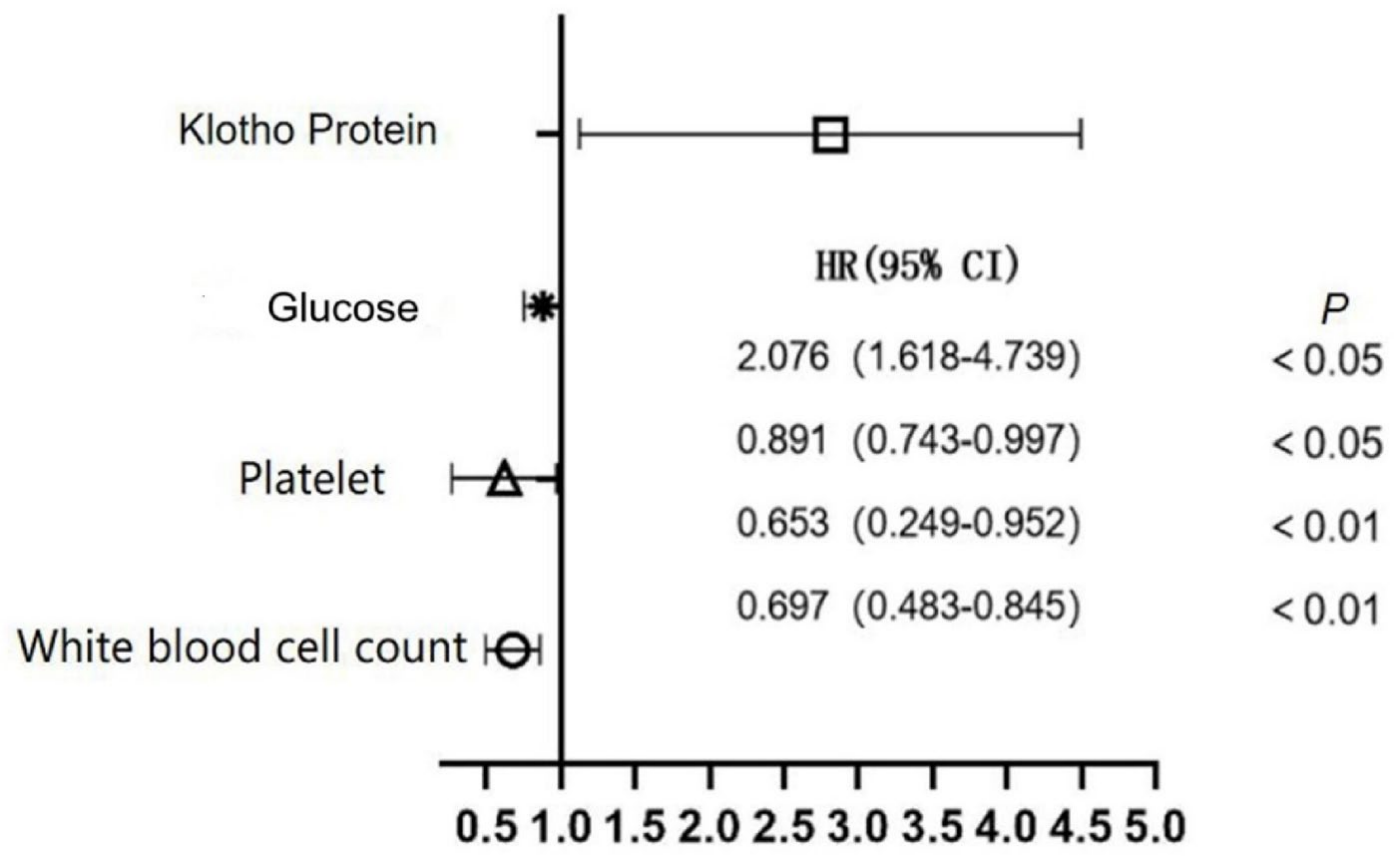
Injury to the brain: multivariate logistic regression analysis of a forest diagram.

Baseline data and serum Klotho protein levels were compared. According to the findings, patients with AKI had lower serum levels of Klotho protein than those without AKI, yet still higher than levels observed in the healthy control group. All of this is illustrated in the following Table 1 and Fig. 1were subjected to the multivariate logistic regression analysis. The variables with *P*-values less than 0.05 between the group of AKI and non-AKI were included in the multivariate logistic regression. White blood cells, platelet counts, and glucose levels were revealed by multivariate logistic regression analysis as independent risk factors for AKI, Klotho protein level was a protective factor (Fig. 1).

**Table 1:** Baseline characteristics table.

Variables	Control	Total	AKI(*n* = 37)	NAKI (*n* = 85)	t/Z/X²	*P*
Age	43(35–48)	49 (40–45)	49(39.3–54.8)	48(39.5–56.5)	-0.162	> 0.05
Gender (men/%)	71(71%)	85(69.7%)	16 (18.8%)	69 (81.2%)	0.510	> 0.05
BMI	23.7 ± 1.38	24.3 ± 3.2	25.1 ± 1.9	22.7 ± 4.1	4.437	< 0.01
MAP (mmhg)	NA	77.62 ± 9.74	65.06 ± 10.13	74.43 ± 9.18	-5.021	< 0.01
Shock	NA	71(58.2%)	20(54.1%)	51 (60%)	0.375	> 0.05
GCS score	NA	6.21 ± 3.5	4.26 ± 2.3	6.31 ± 3.7	-3.718	< 0.01
WBC (10⁹/L)	NA	10.59 ± 4.03	11.54 ± 3.42	9.32 ± 4.18	2.828	< 0.01
Neutrophil (10⁹/L)	NA	13.5(10.7,16.2)	15(12.1,18.4)	13 (10.5–15.7)	2.254	< 0.05
Platelets (10¹²/L)	NA	96(84–108)	86(78–96)	97 (88–113)	-3.535	< 0.01
Lymphocytes (10⁹L)	NA	0.61(0.45,0.89)	0.54(0.37,0.71)	0.65 (0.50–0.92)	2.775	< 0.01
HB (g/L)	NA	107(96.75–1116)	108(98–106)	106 (96–116)	-0.343	> 0.05
Albumin (mmol/L)	NA	31.52 ± 7.76	28.72 ± 7.32	34.25 ± 7.23	-3.834	< 0.01
Glucose (mmol/L)	NA	12.4(10.8–14.2)	13.8(11.9–15.4)	11.8 (10.8-13.75)	-2.756	< 0.01
BUN (mmol/L)	NA	6.4 ± 3.9	7.6 ± 4.9	5.9 ± 4.3	1.923	> 0.05
Creatinine (umol/L)	NA	75.24 ± 16.38	94.1 ± 22.8	64.27 ± 19.52	7.355	< 0.01
Klotho proteins (pg/ml)	134.8(125.6,138.9)	177.3(165.43,195.07)	166.6(149.4,175.3)	192.2 (184.5-192.5)	3.422	< 0.05
APACHE II score	NA	26 (21–29)	28 (24–36)	25 (21–28)	-2.682	< 0.01
90 GOS score	NA	2 (1–2)	1 (1–2)	2 (1–3)	-2.283	< 0.01
In-hospital Mortality rate%	NA	48 (39.3%)	22 (59.5%)	26 (30.6%)	9.004	< 0.01
ICU length of stay (h)	NA	106 (74.8–132)	129 (109–145)	96 (65–126)	3.824	< 0.01

**Fig. 2:**
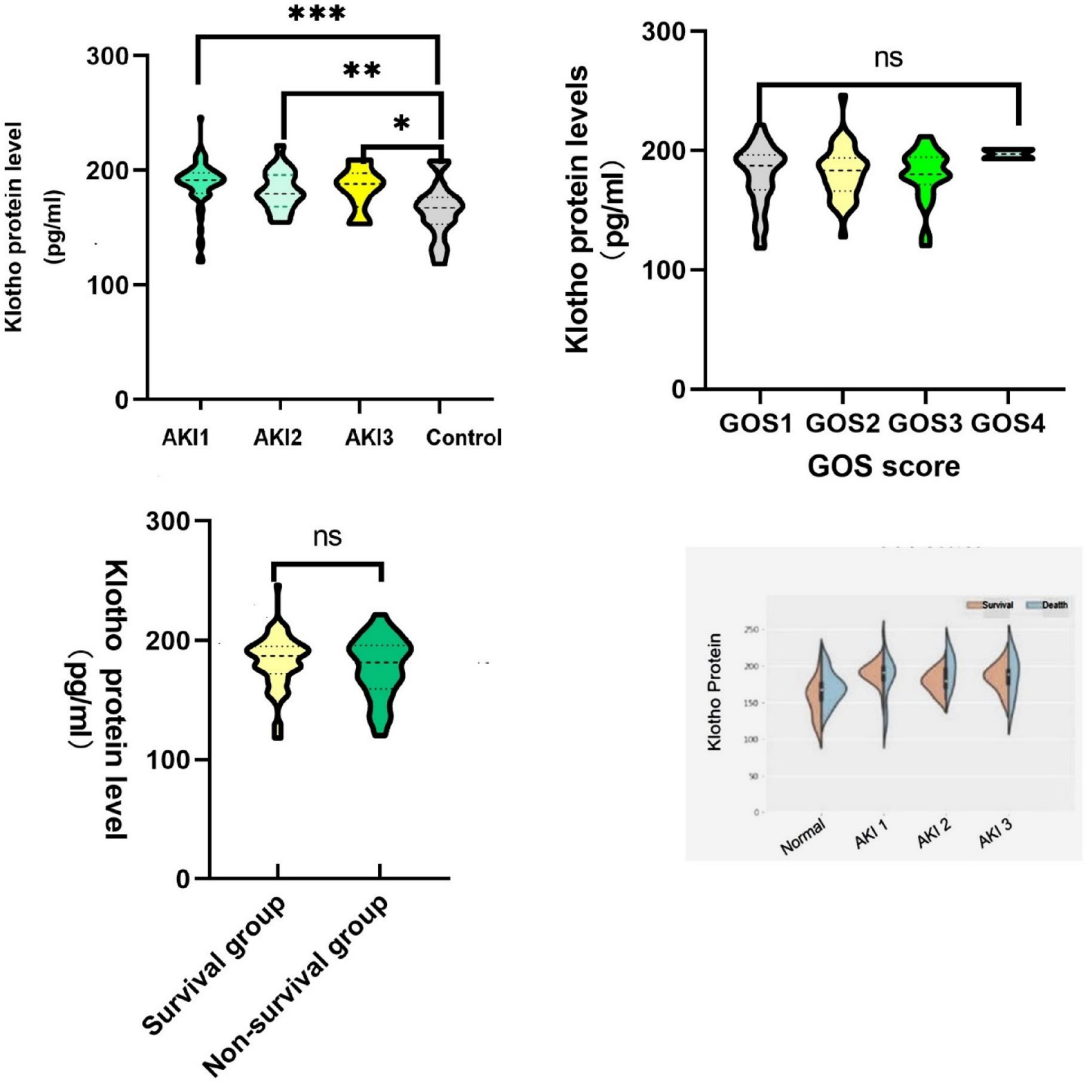
Comparison of the predictive value of serum creatinine and Klotho protein.

**Fig. 3:**
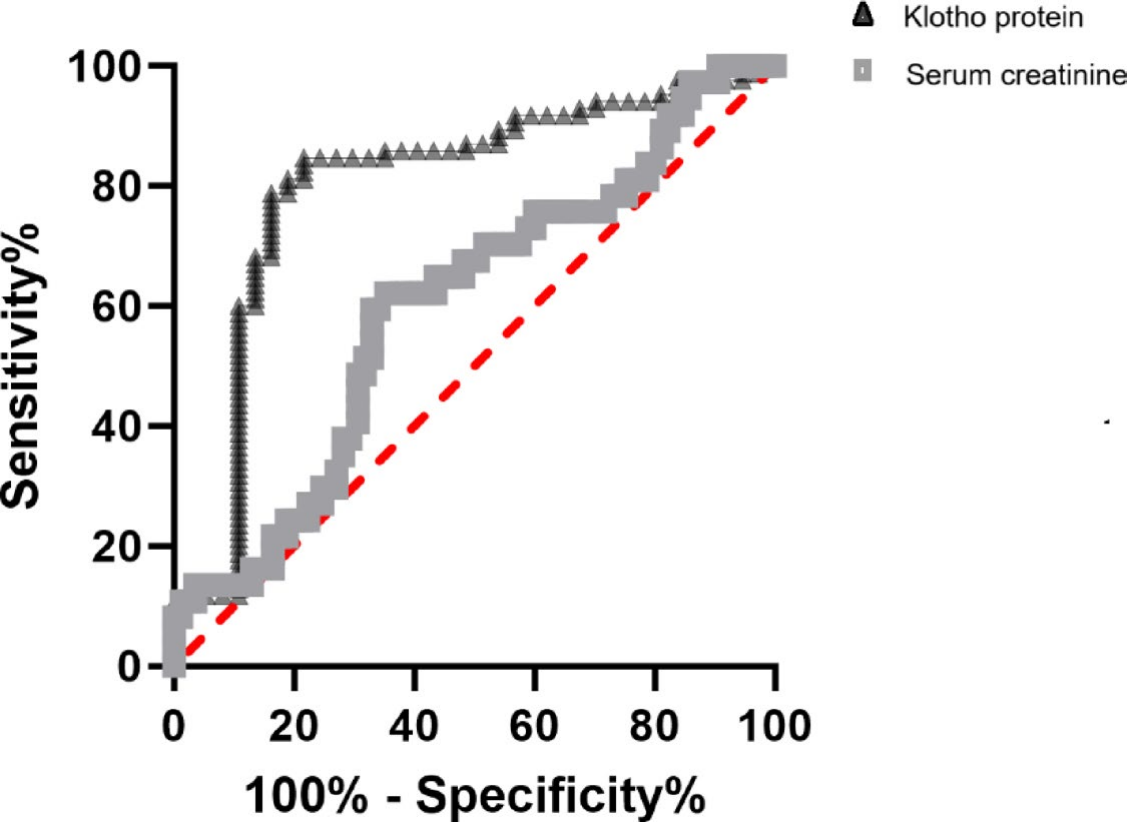
Effect of different Klotho protein levels on prognosis.

**Fig. 4:**
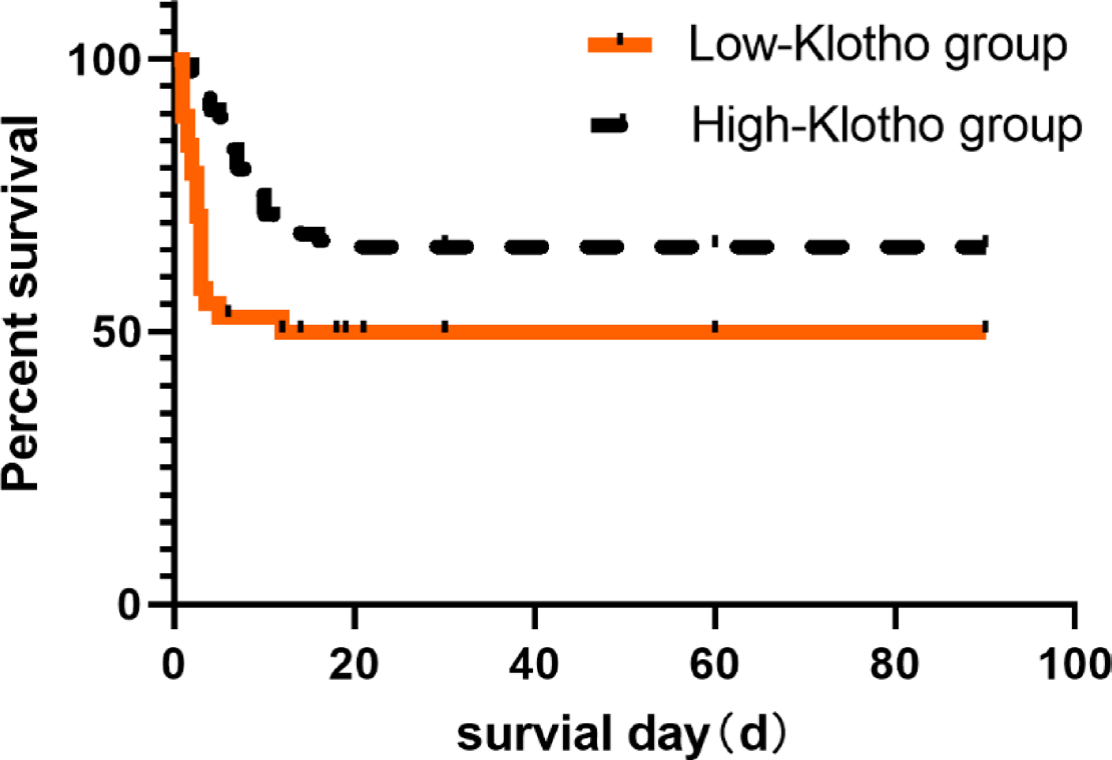
Comparison of GOS scores, survival rates, and groups with various renal functioning for Klotho protein levels.

We demonstrate that Klotho protein had a greater predictive value for AKI than serum creatinine [0.832 (95% CI: 0.709–0.898) vs. 0.6 (95% CI: 0.491–0.709)] (Fig. 2). The cut-off points that predicted mortality in the ROC curve using the Youden index for Klotho protein was ≥ 176.38 pg/ml (sensitivity 84.7% and specificity 78.4%).Then we divided all patients into two groups, lower level group and higher level group according to the cut-off value. The Kaplan-Meier survival curves, depicted in Fig. 3, exhibited patients with lower level klotho protein had lower survival rates. The study also examined the changes in serum Klotho protein levels between various renal function groups, GOS score groups, and survival outcome groups and found that there were sizable variations in Klotho protein levels between various degrees of AKI (Fig. 4). The distribution of serum Klotho protein levels between the groups with normal renal function and the groups with different stages of acute kidney injury was not all the same among the four groups, and post hoc two-by-two comparisons using Bonferroni’s method corrected for the level of significance revealed that the difference in the distribution of serum Klotho protein levels was statistically significant only between the groups with normal renal function and the two groups with stage 1 AKI (adjusted Z=-4.942, *P* < 0.001), and the remaining two-by-two comparisons showed no statistically significant differences (Fig. 4; Table 2); the differences in the distribution of serum Klotho protein between the different GOS score groups (Table 3) and the different survival outcome groups were not statistically significant (H = 2.624, *P* = 0.353; Z=-0.812, *P* = 0.417; Figs. 4), and the differences in the distribution of serum Klotho protein between the different survival outcome of the group with normal renal function, and serum Klotho protein distribution in the group with different stages of acute kidney injury had no group differences [(Z=-1.896, *P* = 0.058; Z=-0.771, *P* = 0.441; Z=-1.429, *P* = 0.153; and Z=-0.342, *P* = 0.732); Fig. 4; Table 4].

**Table 2:** Intergroup comparison of serum Klotho protein in different renal function stages.

Groups	Test statistic	Standard error	Standard test statistic	*P* value	Adjusted *P* value
Control vs. AKI 1	-37.518	7.591	-4.942	<0.001	<0.001
Control vs. AKI 2	-25.707	10.308	0.013	0.013	0.08
Control vs. AKI 3	-29.318	12.721	0.021	0.021	0.127
AKI 1vs AKI 2	12.011	9.503	0.206	0.206	1
AKI 1vs AKI 3	8.200	12.079	0.497	0.497	1
AKI 2vs AKI 3	-3.811	13.947	0.785	0.785	1

**Table 3:** GOS subgroups.

	Number	Percentage	Effective percentage	Cumulative percentage
GOS 1	59	48.4	48.4	48.4
GOS 2	36	29.5	29.5	77.9
GOS 3	25	20.5	20.5	98.4
GOS 4	2	1.6	1.6	100.0

**Table 4:** Intergroup comparison of serum Klotho protein for different survival outcomes in different renal function states.

Statistic	Control	AKI 1	AKI 2	AKI 3
Z	-1.896	-0.771	-1.429	-0.342
P	0.058	0.441	0.153	0.732

## Discussions

Traumatic brain injury (TBI) constitutes a major global health burden, with mortality rates significantly higher in patients developing AKI as a complication. Current AKI diagnosis and staging mainly rely on the 2012 Kidney Disease: Improving Global Outcomes (KDIGO) criteria, which employ serum creatinine (SCr) and urine output (UOP) as primary parameters. However, SCr exhibits critical limitations as a diagnostic biomarker^20^: Its delayed elevation reflects functional glomerular filtration rate (GFR) decline rather than early structural renal injury. Concurrently, UOP assessment faces diagnostic ambiguity due to diuretic administration and prerenal azotemia.Preclinical studies underscore that therapeutic interventions for TBI-associated AKI require initiation during the subclinical phase, preceding detectable SCr changes, to mitigate irreversible renal damage. These findings highlight an urgent need for novel biomarkers capable of detecting AKI at incipient stages, enabling timely and targeted management.

The Klotho gene is closely associated with the diagnosis and treatment of acute kidney injury (AKI). It not only holds promise as an early diagnostic biomarker for AKI but also represents a potential therapeutic target. Preclinical studies have demonstrated that serum Klotho protein levels are significantly reduced in rat models of AKI, while exogenous Klotho supplementation exhibits renoprotective effects^21^.This study revealed that traumatic brain injury induces elevated serum Klotho protein levels, yet the defenite mechanisms are still ambiguous due to studies on serum Klotho andTBI are scarce.Interestingly, patients with TBI complicated by AKI exhibited significantly lower Klotho level compared to those without AKI. This phenomenon may be attributed to the kidney being a major site of Klotho protein expression, which serves as a primary source of circulating Klotho protein^22^. During AKI, elevated Klotho levels are hypothesized to exert renoprotective effects through multiple mechanisms, including suppression of inflammatory responses and enhancement of autophagy activity^23^.

Serum Klotho protein levels may alter before major creatinine abnormalities in AKI, according to animal investigations^24^. The potential of Klotho protein as a sensitive biomarker for early AKI detection can be shown from the fact that its diagnostic accuracy was higher than that of serum creatinine^25^ Consistent with previous studies, our cohort analysis also demonstrated the same results.These results, however, require confirmation in larger clinical investigations.

The serum Klotho protein level independently operated as a protective factor against AKI in individuals with craniocerebral injury, according to the multivariate analysis in this study. Further investigation revealed that, whereas long-term prognosis was influenced by Klotho protein levels, short-term survival results were not. These results were consistent with reports of the anti-inflammatory and vasodilatory effects of Klotho^26^.

According to the study’s findings, serum Klotho protein levels can help with the early detection and prognosis of AKI in those who have suffered a craniocerebral injury. For directing early intervention and improving treatment plans, this information is helpful. Klotho protein levels can also be used as a monitoring indicator for determining the prognosis over the long run. Although we initially explored the early predictive value of serum Klotho protein levels, there were limitations to the experiment; first, our patients were from a single-center sample, and second, due to the primary nature of the study’s objectives, we did not explore the impact of serum Klotho protein, a more inflammatory indicator. However, more research, including bigger patient cohorts and multi-center partnerships, is required to explore the processes driving various clinical outcomes due to the complex physiological changes following traumatic brain injury, and the effects of multiple inflammatory markers on serum Klotho protein in the context of inflammation and oxidative stress.

In summary, serum Klotho protein levels may help with prognosis prediction and early AKI diagnosis in individuals with traumatic brain injury. This knowledge can improve patient care in general and direct early actions. Monitoring Klotho protein levels can also reveal information about a patient’s long-term prognosis. To further our understanding of the clinical outcomes in patients with craniocerebral damage, more research with larger sample sizes, multi-center studies, and fundamental experiments is required.

## Conclusions

Our study showed that serum Klotho protein has the potential to be a useful biomarker for the early detection and prognosis of acute kidney injury (AKI) in patients with craniocerebral injuries. serum Klotho protein levels may be more sensitive in recognizing AKI than more established markers such as blood creatinine. The study also highlighted the serum Klotho protein’s protective role in AKI since it works on its own to prevent AKI from developing in those who have had a craniocerebral injury. A larger patient cohort and multi-center partnerships are required for future research to validate these findings and further explore the underlying mechanisms because of the small sample size of this study. Overall, the discovery of serum Klotho protein as a possible biomarker for the early detection of AKI can be used to direct early interventions, improve treatment plans, and offer important insights into the long-term prognosis of these individuals.

## Data Availability

Data will be made available on request. However, we encourage researchers with legitimate research purposes who wish to access these data to contact us using the following method to request data access.Please contact Bin Ren, Email: renbinlc@126.com, and provide a detailed research plan along with a specific description of how the requested data will be used. We will evaluate each request on a case-by-case basis and strive to provide support or offer appropriate alternatives as feasible.Please note that all data sharing will comply with applicable laws, ethical guidelines, and institutional policies.

## Declarations

### Author agreement

All authors have seen and approved the final version of the manuscript being submitted. They warrant that the article is the authors’ original work, hasn’t received prior publication and isn’t under consideration for publication elsewhere. The manuscript meets all the authors’ instructions.
